# DRACP: a novel method for identification of anticancer peptides

**DOI:** 10.1186/s12859-020-03812-y

**Published:** 2020-12-16

**Authors:** Tianyi Zhao, Yang Hu, Tianyi Zang

**Affiliations:** Department of Computer Science and Technology, School of Life Science and Technology, Harbin Institute of Technology, Harbin, China

**Keywords:** Anticancer peptides, Deep belief network, Relevance vector machine, Random forest, Cancer

## Abstract

**Background:**

Millions of people are suffering from cancers, but accurate early diagnosis and effective treatment are still tough for all doctors. Common ways against cancer include surgical operation, radiotherapy and chemotherapy. However, they are all very harmful for patients. Recently, the anticancer peptides (ACPs) have been discovered to be a potential way to treat cancer. Since ACPs are natural biologics, they are safer than other methods. However, the experimental technology is an expensive way to find ACPs so we purpose a new machine learning method to identify the ACPs.

**Results:**

Firstly, we extracted the feature of ACPs in two aspects: sequence and chemical characteristics of amino acids. For sequence, average 20 amino acids composition was extracted. For chemical characteristics, we classified amino acids into six groups based on the patterns of hydrophobic and hydrophilic residues. Then, deep belief network has been used to encode the features of ACPs. Finally, we purposed Random Relevance Vector Machines to identify the true ACPs. We call this method ‘DRACP’ and tested the performance of it on two independent datasets. Its AUC and AUPR are higher than 0.9 in both datasets.

**Conclusion:**

We developed a novel method named ‘DRACP’ and compared it with some traditional methods. The cross-validation results showed its effectiveness in identifying ACPs.

## Background

In recent decades, the number of cancer patients has always been increasing. The elder people concerned more on cancers than neurodegenerative diseases. Although the rapid development of medical technology helps a lot, the death rate of patients and burden of the society are still very high. The traditional methods such as radiation therapy [1], targeted therapy [2] and chemotherapy [3] can help suppress cancers, but apart from the expensive cost, the harm of these treatments to patients are unmeasured [4]. Apparently, finding a unharmful treatment for cancers is critical.

In 1972, antimicrobial peptides’ primary structure have been found by Boman [5]. Following his research, many researchers found these peptides have antitumor activity [6, 7]. Therefore, these antimicrobial peptides were named as anticancer peptides (ACPs). ACPs not only have the advantages of high specificity and high tumor penetration, but also easy to synthesis and unharmful to normal cells [8]. This significant advantage makes ACPs become the most potential treatment for cancers [9, 10].

Most of the ACPs are combined from 12–50 amino acid residues. Many of these ACPs’ structure are α-helical or β-sheet and some special ACPs have particular folds. They execute their function by interacting with the anionic cell membrane components of cancer cells and then selectively kill cancer cells [11, 12]. Most of the ACPs are obtained from Antimicrobial peptides (AMPs) [13] since cationic AMPs destroy only bacteria but not the normal cells, which shows a broad spectrum cytotoxicity against various cancer cells [14]. Although the mechanism of ACPs is not fully clear at present [15, 16], the development of natural ACPs and artificially designed peptides are still important ways to against cancer.

Due to the high cost of money and time in finding ACPs, increasing number of researchers have focused on identifying the ACPs by computing method. Tyagi et al. [17] extracted amino acid composition and binary profiles as features to build a SVM model to identify ACPs. Later, Khosravian et al. [18] also used SVM to find the ACPs. Then, Hajisharifi et al. [19] used the same method to identify the ACPs, with Chou’s pseudo amino acid composition. Besides, Chen et al. [20] purposed a new method named IACP to find ACPs, which has made a great progress. Recently, Manavalan et al. [21] used both Random Forest and SVM to identify the ACPs. Felício et al. [7] reviewed the development of ACPs in 2017 and pointed ACPs decreases the probability of resistance and discussed the relationship between AMP and ACP. Grisoni et al. [22] used long short-term memory (LSTM) to identify ACPs based on sequence.

Although these methods play an important role in the development of this area, there still need more complex algorithm to achieve higher accuracy. Biological networks are common methods to identify biological molecule [23]. In recent years, deep learning algorithms have been widely used in bioinformatics field [24–27]. Deep belief network (DBN) has been proven to be a powerful tool to encode [28]. Therefore, we purposed a novel method named DRACP to identify ACPs. To verify the effectiveness of our method, we used the method on two different datasets. For each, we did cross-validation to do the test to verify the stability.

## Results

### Data description

The datasets of ACPs was downloaded from Wei Chen et al. [20]. We obtained two datasets. One of them contains 138 real ACPs samples and 206 non-ACPs samples. The other one has 150 real ACPs samples and 150 non-ACPs samples. All the negative samples are randomly generated.

In this paper, 10-cross validation was used to test our method, that is, dividing the whole dataset into 10 groups and one of the groups is used as testing dataset and the rest of groups are used as training dataset.

### The performance of DRACP compared with previous method

In this study, the label of pseudo ACPs is 0, and the label of real ACPs is 1.

Firstly, we executed DRACP on the two datasets. The average accuracy of first dataset is 86.87% and the number is 85.17% for the second dataset.

Tyagi et al. [17] developed a method for identifying ACPs based on SVM. We compared our method with their method.

Compared with Tyagi et al. method, we used different features and method. Although different features are used by Tyagi et al., their best performance one is dipeptide composition-based SVM model. However, they ignored the chemical characteristics of amino acids. To test the importance of our feature, we also built a SVM model by using our features. We called this method SVM_NF_.

The performance these three methods are shown in Table 1. As shown in Table 1, DRACP performed best among these method with the accuracy 0.96 and 0.95. SVM_NF_ ranked second, which means our features are better than Tyagi et al.’s.

**Table 1:** The accuracy of three methods

	Dataset 1	Dataset 2
DRACPs^a^	0.96	0.95
SVM_NF_^b^	0.92	0.91
Tyagi et al^c^	0.88	0.86
Naive Bayes	0.84	0.81
Random forest	0.89	0.85

^a^The method we purposed

^b^SVM with our feature

^c^Available at https://crdd.osdd.net/raghava/anticp/multi_pep.php

### The necessity of using DBN

Without using DBN, we put 56-dimension features into Random Relevance Vector Machines (RRVMs) to built the model. Same testing method was used to compare the performance of DRACP and RRVMs. This time, AUC and AUPR are used to evaluate the accuracy of classification.

**Fig. 1:**
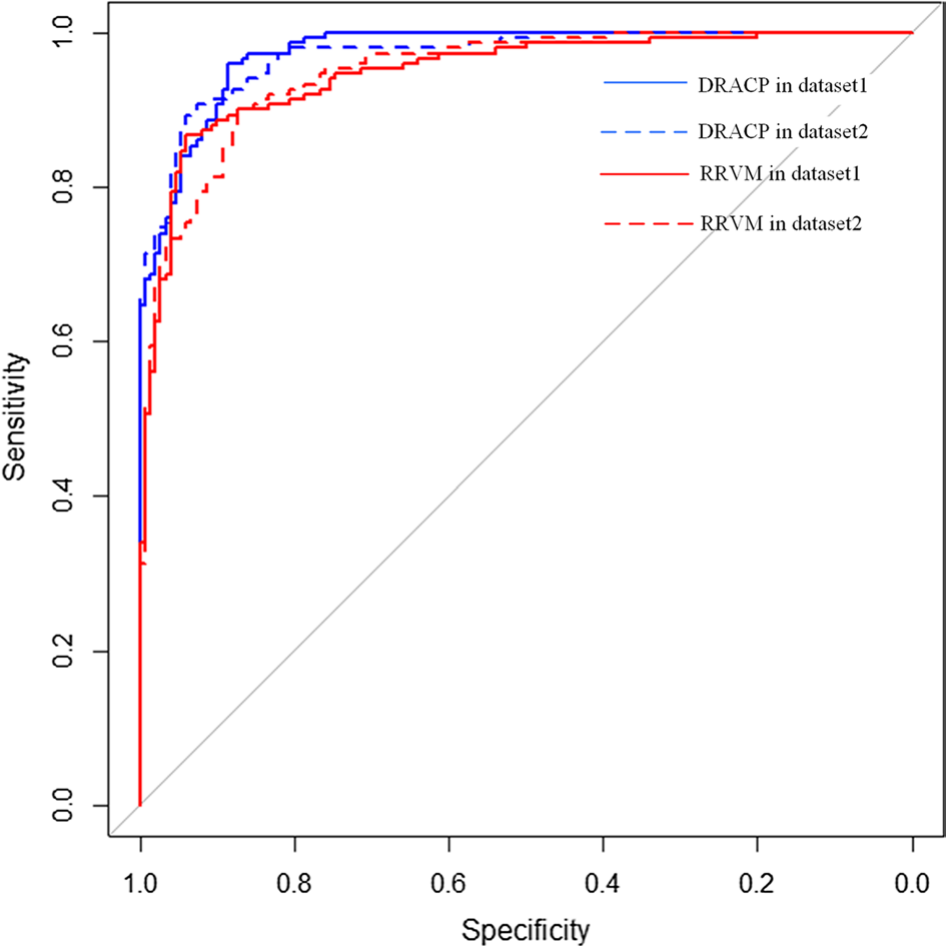
The ROC curves of DRACP and RRVMs

Figure 1 shows the ROC curves of DRACP and RRVMs. The blue lines denote the ROC curves of DRACP and the red lines denote the ROC curves of RRVMs. The results of dataset2 are represented by dotted lines and solid lines for the results of dataset1. As we can see, DRACP performed much better than RRVMs. Then, we tested the AUPR of these two methods.

**Fig. 2:**
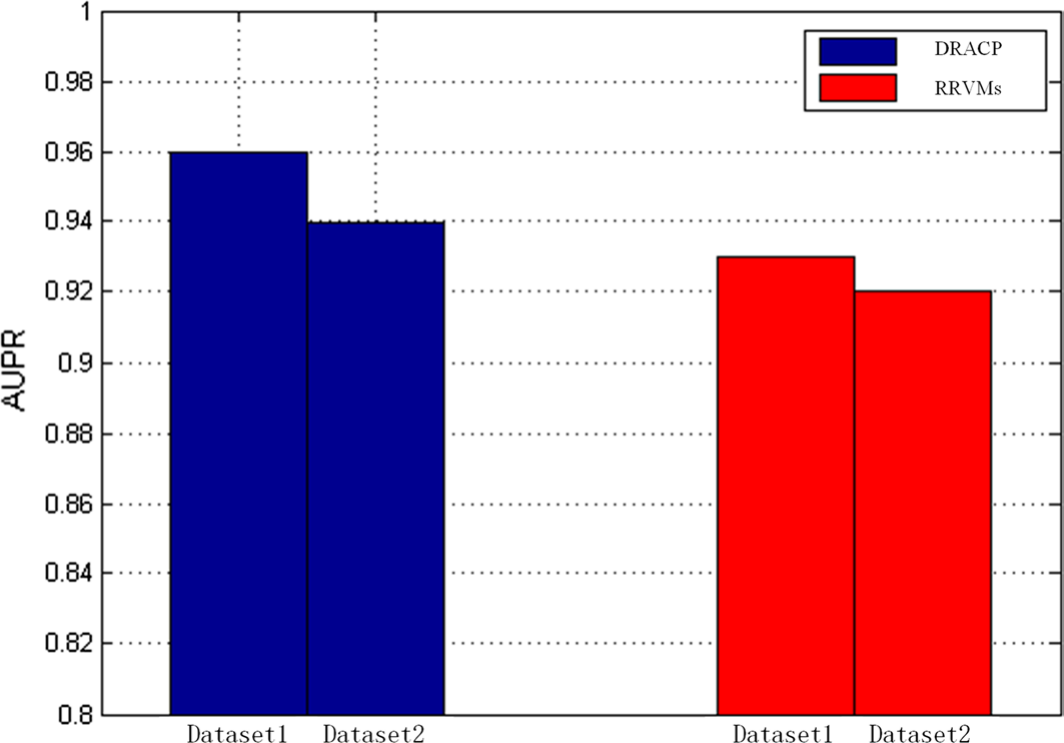
The PR curves of DRACP and RRVMs

Figure 2 shows the PR curves of DRACP and RRVMs. The blue bars denote PR curves of DRACP and red bars denote PR curves of RRVMs. DRACP performed better than RRVMs too.

These experiments showed that using DBN to encode could improve the accuracy of the model.

## Discussion

Most of the previous methods for identifying ACPs are based on the traditional methods such as SVM. As the development of algorithms, more powerful methods should be applied into identifying ACPs.

In this paper, we used DBN to encode the feature of ACPs. DBN reduces dimension of ACP features through unsupervised learning. Then, we developed RRVMs which is a method based on RVM and RF to identify true ACPs. The experiments showed high precision of DRACP, which verified DRACP is an effective method for identifying ACPs. In addition, we also show the power of DBN by comparing the results of DRACP with RRVM’s. This experiment explained the necessity of reducing dimension of features by DBN. Finally, we compared our method with previous methods and some traditional methods to show the superiority of DRACP.

DRACP can prior the potential ACPs based on their sequence. This work will help biologist reduce the cost of money and time on finding ACPs.

## Conclusions

With its harmless advantages to the human body, ACPs have a great potential for treating cancers. However, due to the high cost of finding ACPs, not many ACPs have been found and there is still long way to go to use ACPs as a treatment.

To reduce the cost of money and time for finding ACPs, in this study, we proposed a method named DRACP to identify ACPs based on sequence and chemical characteristics of amino acids. Since the dimension of each ACP’s feature is high, DBN was used to encode the features in a unsupervised way. It can effectively reduce the dimension and keep the information of features. After obtaining the final features, we randomly selected features and samples to build RVM models. 101 RVM models were built to generate a final classifier. This building process draw the idea of RF.

To verify the performance of DRACP, we use two independent datasets with 10-cross validation to do the test. We not only proved the performance of DRACP was better than previous method, but also showed the power of our features. In addition, we also test the performance of using RRVM without DBN and found DBN is an essential part for improving accuracy.

Overall, we developed an effective method for identifying ACPs. Although our method performed well, larger datasets are still needed to further prove the power of DRACP.

## Methods

### Feature extraction

#### Compositional analysis

**Fig. 3:**
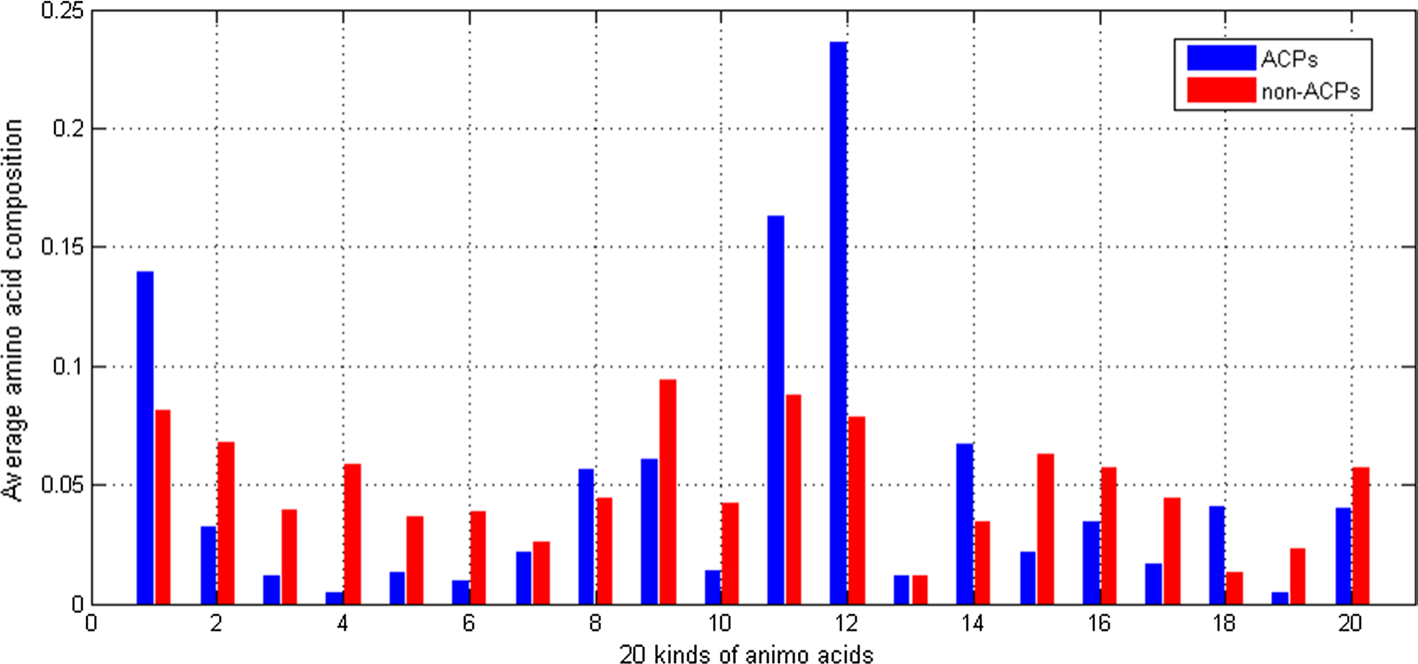
Comparison of average whole amino acids composition of ACPs and non-ACPs. x-axis is the index of 20 kinds of amino acids and y-axis is the ratio of the amino acid to the total sequence length

We conjectured the composition of real ACPs are different from other normal peptides. Therefore, the average percentage of each amino acid is shown in Fig. 3.

As shown in Fig. 3, the blue bar denotes the composition of real ACP and the red one is the non-ACPs’. Among the 20 amino acids, only 3 amino acids almost share the same percentage. Most of the composition of amino acids have significant differences between ACPs and non-ACPs. Therefore, the composition of 20 amino acids could be the features of ACPs.

#### The reduced amino acid composition

Protein structure is closely related to the patterns of hydrophobic and hydrophilic residues. The amino acids are divided into 6 groups based on the ranges of the hydropathy scale. Table 2 shows the six groups of the 20 amino acids.

**Table 2:** The six groups of the 20 amino acids

Groups	Amino acids
Strongly hydrophilic	R, D, E, N, Q, K, H
Strongly hydrophobic	L, I, A, V, M, F
Weakly hydrophilic Weakly hydrophobic	S, T, Y, W
Proline	P
Glycine	G
Cysteine	C

Therefore, we can use six characters to represent the sequence of peptides. Since the dipeptides are consisted by two peptides, there would be $6^{2}$ features to describe a sequence. Then we could extract the feature as following:

1 $$F = [f_{1} ,f_{2} ,f_{3} \ldots f_{36} ]$$

where $f_{x}$ is the absolute occurrence frequencies of the 36 hydropathy dipeptides. It can be calculated as following:

2 $$f_{i} = \frac{{n_{i} }}{L - 1}$$

where $n_{i}$ is the occurrence number of the 36 hydropathy dipeptides of the protein, L is the length of peptide.

**Fig. 4:**
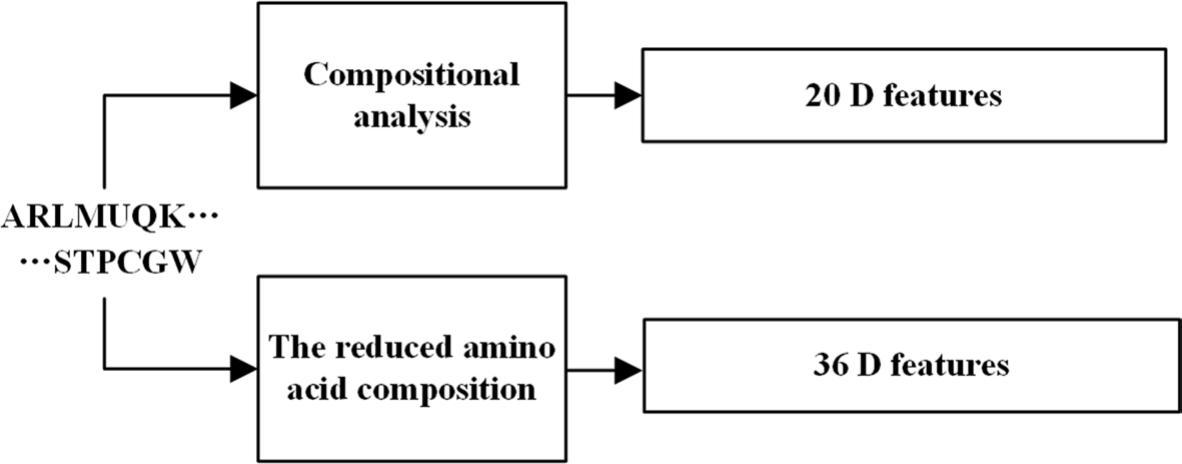
Flow chart of feature extraction. We extracted 56 D features to identify the ACPs and it includes 20-dimensional composition and 36-dimensional reduced amino acid composition

The Fig. 4 shows the flow chart of feature extraction. In total, we extracted 56 D features to identify the ACPs.

### Methods and framework

**Fig. 5:**
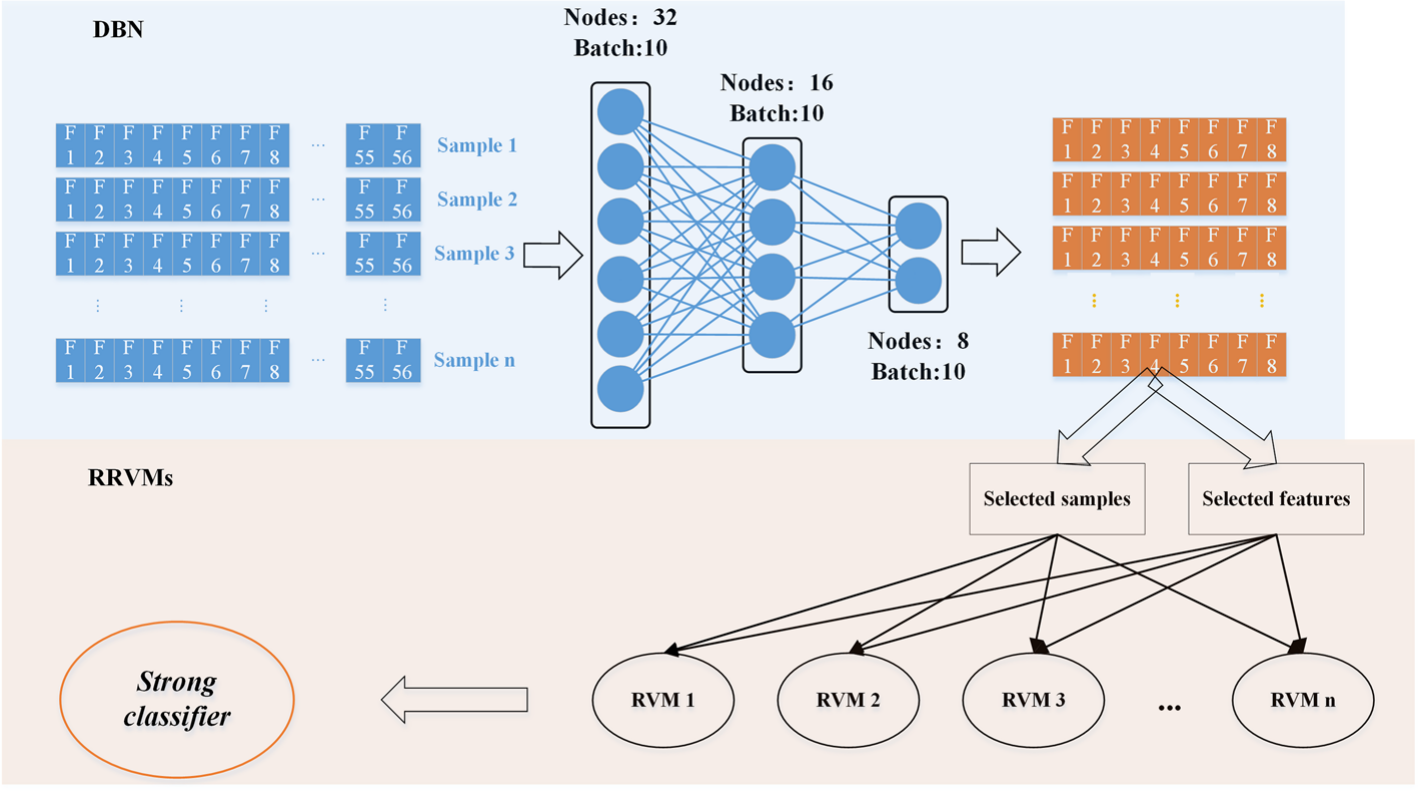
Frame of DRACP. The first step is to use DBN to reduce the dimension of features. Then RRVMs is used to do classification

Firstly, DBN was used to encode the features we obtained above. Then RRVM was used to classify ACPs. The workflow of our method is shown in Fig. 5.

#### DBN

DBN is an efficient semi-supervised algorithm. A layer-by-layer greedy algorithm is used to train the parameters of the deep belief network, breaking the deadlock that has been difficult for deep networks for a long time.

Restricted Boltzmann Machine (RBM) is the basic unit of DBN. The variables in RBM are divided into hidden variables and observable variables. These two sets of variables are represented by observable and hidden layers, respectively. There is no connection between nodes in the same layer, and nodes in one layer are connected to all nodes in another layer, which is as same as the fully connected neural network structure.

An RBM is composed of m observable variables and n hidden variables, and its energy function is defined as:

3 $$E(v,h) = - \sum\limits_{i} {a_{i} v_{i} - } \sum\limits_{j} {b_{j} h_{j} - } \sum\limits_{i} {\sum\limits_{j} {v_{i} w_{ij} h_{j} = } } - a^{T} v - b^{T} v - v^{T} Wh$$

Here, v is an observable variable $v = [v_{1} ,v_{2} ,...v_{m} ]^{T}$ and h is a hidden random vector $h = [h_{1} ,h_{2} ,...h_{n} ]^{T}$. W is a weight matrix, its dimension is m * n, and each element is the weight of the edge between the observable variable and the hidden variable. Both a and b are biases, a is the bias of the observable variable v, and b is the bias of the hidden variables.

The joint probability distribution of RBM is $p(v,h)$ which could be calculated by:

4 $$p(v,h) = \frac{1}{Z}\exp ( - E(v,h)) = \frac{1}{Z}\exp (a^{T} v)\exp (b^{T} h)\exp (v^{T} Wh)$$

where $Z = \sum\limits_{v,h} {\exp ( - E(v,h))}$ is the partition function.

The essence of DBN is the stacking of RBMs. For a DBN containing L-level hidden variables, the lowest level is $v = h^{(0)}$ which is the observable variable. The top two layers are an undirected graph used to generate the prior distribution of $p(h^{(L - 1)} )$. Except for the top two layers, each layer can be calculated by the layer above it:

5 $$p(h^{(l)} |h^{(l + 1)} ,...,h^{(L)} ) = p(h^{(l)} |h^{(l + 1)} )$$

The joint probability of variables in DBN can be denoted by:

6 $$p(v,h^{(1)} ,...,h^{(L)} ) = p(v|h^{(1)} )\left( {\prod\limits_{l = 1}^{L - 2} {p(h^{(l)} |h^{(l + 1)} )} } \right)p(h^{(L - 1)} ,h^{(L)} )$$

where $p(h^{(l)} |h^{(l + 1)} )$ is sigmoid conditional probability distribution.

#### RRVMs

We learnt the basic idea from random forest (RF) to propose a new method RRVMs. By randomly selecting features and samples, RVM was built as a weak classifier. We repeated this process 101 times to construct a strong classifier.

First, we randomly select 5 features and 100 samples to build up a RVM model. Then, we put these features and samples back and started another round of building model. This process could be repeated 101 times, so 101 RVM models would be obtained. In the end, the strong classifier could be obtained by getting votes from these 101 RVM models.

#### The construction of RVM classifier

Compared with Support vector machine (SVM), the kernel function of RVM is not limited by Mercer conditions. It could be more sparse and has less super-parameters, so it reduces the computational burden of kernel functions.

For a given dataset $\{ x_{i} ,t_{i} \}_{i = 1}^{N}$, $x_{i} \in {\text{R}}^{d}$, non-linear model is :

7 $$t = y(x) + \varepsilon$$

where N is the sample number, y() is the non-linear function, $\varepsilon$ is the noise, $\varepsilon \sim N(0,\sigma_{{}}^{2} )$.

The final function of RVM is:

8 $$t = {{\varvec{\Phi}}}\omega + \varepsilon$$

where $\omega = (\omega_{0} , \ldots ,\omega_{N} )^{{\text{T}}}$ is the weight, ${{\varvec{\Phi}}}$ is the matrix of the kernel function. K() is the kernel function.$\phi_{i} (x_{i} ) = \left[ {1,K(x_{i} ,x_{1} ), \ldots ,K(x_{i} ,x_{N} )} \right],i = 1,2, \ldots ,N$.

The distribution of $p(t|x)$ meets $N(t|y(x),\sigma^{2} )$. Likelihood estimation of data is:

9 $$p(t|\omega ,\sigma^{2} ) = (2\pi \sigma^{2} )^{ - N/2} \exp \{ - \left\| {t - {{\varvec{\Phi}}}\omega } \right\|^{2} /(2\sigma^{2} )\}$$

Tipping defines a zero mean Gauss type prior distribution on $\omega$:

10 $$p(\omega /\alpha ) = \prod\limits_{0}^{N} {N(\omega_{i} |0,\alpha_{i}^{ - 1} )} = \prod\limits_{0}^{N} {\frac{{\alpha_{i} }}{{\sqrt {2\pi } }}\exp \left( {\frac{{\omega_{i}^{2} \alpha_{i} }}{2}} \right)}$$

where $\alpha$ is the super-parameter, it is one-to-one correspondence to the weight.

$\alpha$ and the variance of noise $\sigma^{2}$ meet the Gamma distribution.

11 $$\begin{aligned} & p(\alpha ) = \prod\limits_{i = 0}^{N} {{\text{Gamma}}(\alpha_{i} |a,b)} \\ & p(\sigma^{2} ) = \prod\limits_{i = 0}^{N} {{\text{Gamma}}(\beta |c,d)} \\ \end{aligned}$$

When there is a new set of observations, the prediction based on the sparse Bayesian learning framework can be expressed as:

12 $$p(t_{N + 1} |t) = \int {p(t_{N + 1} |\omega ,\alpha ,\sigma^{2} )} p(\omega ,\alpha ,\sigma^{2} |t)d\omega d\alpha d\sigma^{2}$$

where $t_{N + 1}$ is the target value of the new observation $x_{N + 1}$.

For a new set of inputs $x_{*}$, the output $t_{*}$ should meet the distribution $p(t_{*} |t)\sim N(\mu^{{\text{T}}} {{\varvec{\Phi}}}(x_{*} ),\sigma_{*}^{2} )$.

13 $$t_{*} = \mu^{{\text{T}}} {{\varvec{\Phi}}}(x_{*} )$$

14 $$\sigma_{*}^{2} = \sigma_{MP}^{2} + {{\varvec{\Phi}}}(x_{*} )^{{\text{T}}} \sum {{\varvec{\Phi}}}(x_{*} )$$

where $\sigma_{MP}^{2}$ is the final variance of noise.

To accomplish the construction of classifier, we also need to set the various parameters as Table 3 shows.

**Table 3:** Parameters and functions of RVM

Setting items	The value set
Max iterations	100
Kernel function	Gaussian
Kernel function width	6
Sample number	50
Feature number	10

## Data Availability

All the datasets used in this paper could be downloaded from https://github.com/zty2009/ACP.

## Abbreviations

ACP: Anticancer peptides
DBN: Deep belief network
AMP: Antimicrobial peptide
RBM: Restricted Boltzmann machine
RF: Random forest
RRVM: Random Relevance Vector Machines
SVM: Support vector machine
LSTM: Long short-term memory
